# Supplementary material for: Content-rich biological network constructed by mining PubMed abstracts
Source: BMC Bioinformatics. 2004 Oct 8;5:147. doi: 10.1186/1471-2105-5-147 (PMC528731; doi:10.1186/1471-2105-5-147)
Supplement: Additional File 2 — The original results of the above study (non-essential files are deleted to keep the file size under the limit set by BMC bioinformatics). [file 1471-2105-5-147-S2.bz2 › chilibotAdditionalFile2/dip05/729ID11333269E18086/html/DNAB_DNAC.html]

 


 **DNAB** and **DNAC** 
  
Found 151 abstracts in PubMed, retrieved 05.  
 

 What does Google say? 
 PDF only 
| .edu only 

---

**Interactive relationship** (e.g. stimulation, inhibition, etc)

**Neutral relationship**- Quantitative studies of interactions between the Escherichia coli replication factor  **DnaC**  protein and the  **DnaB**  helicase have been performed using sedimentation velocity and fluorescence energy transfer techniques.  Ref: 12767828 J Mol Biol, 2003
- Interactions of the Escherichia coli  **DnaB**  Helicase Hexamer with the Replication Factor the  **DnaC**  Protein.  Ref: 12767828 J Mol Biol, 2003
- We show that DnaI and  **DnaB**  interact physically and functionally with the  **DnaC**  helicase and mediate its functional delivery onto DNA.  Ref: 12718886 Mol Cell, 2003
- The data establish that, in the presence of the ATP analog AMP PNP, or ADP, a maximum of six  **DnaC**  monomers bind cooperatively to the  **DnaB**  hexamer.  Ref: 12767828 J Mol Biol, 2003
- Thus, Cdc6 may act as the functional homologue of the bacterial  **DnaC**  that utilizes ATP hydrolysis to assemble the  **DnaB**  helicase at the origin.  Ref: 12837750 J Biol Chem< MedlineTA>J Biol Chem, 2003
- Experiments have been performed with the fluorescein modified  **DnaB**  helicase, which allows an exclusive monitoring of the  **DnaB**   **DnaC**  complex formation.  Ref: 12767828 J Mol Biol, 2003
- In solution, the  **DnaC**  protein binding site is located on the large 33 kDa domain of the  **DnaB**  helicase.  Ref: 12767828 J Mol Biol, 2003
- These data suggest strongly that the  **DnaC**   **DnaB**  complex exists in vivo as a mixture of complexes with a different number of bound  **DnaC**  molecules, although the complex with six  **DnaC**  molecules bound dominates the distribution.  Ref: 12767828 J Mol Biol, 2003
- We also present evidence that the  **DnaC**  helicase loading mechanism appears to be of the ring assembly type, proceeding through the recruitment of  **DnaC**  monomers and their hexamerization around single stranded DNA by the coordinated action of DnaI and  **DnaB** .  Ref: 12718886 Mol Cell, 2003

**Non-interactive relationship** (e.g. studied together, co-existance, homology, etc.)

- These mutations are alleles of genes 32 and 41, which respectively encode a single stranded DNA binding protein gp32 and the replicative DNA helicase  [ **DNAC** ]   [ **DNAB** ]  gp41.  Ref: 12697750 J Biol Chem< MedlineTA>J Biol Chem, 2003
- The significance of the results in the functioning of the  **DnaB**  helicase  **DnaC**  protein complex is discussed.  Ref: 12767828 J Mol Biol, 2003
- The topology of the ternary complex  **DnaC**   **DnaB**  ssDNA has been determined using the fluorescence energy transfer method.  Ref: 12767828 J Mol Biol, 2003
- We report the biochemical characterization of the bacterial hexameric replicative helicase  **DnaC**  of Bacillus subtilis with that of the two replication initiation proteins DnaI and  **DnaB** .  Ref: 12718886 Mol Cell, 2003
- A two protein strategy for the functional loading of a cellular replicative DNA helicase  [ **DNAC** ]   [ **DNAB** ] .  Ref: 12718886 Mol Cell, 2003
- However, in the presence of the DNA, there is a significant difference in the energetics and structure of the ternary complex,  **DnaC**   **DnaB**  ssDNA, formed in the presence of AMP PNP as compared to ADP.  Ref: 12767828 J Mol Biol, 2003
- We propose that the tight MCM Cdc45 complex functions as a replicative DNA helicase  [ **DNAC** ]   [ **DNAB** ]  in vivo.  Ref: 12581157 Genes Cells, 2003
